# Development and Assessment of Herpes Simplex Virus Type 1 (HSV-1) Amplicon Vectors with Sensory Neuron-Selective Promoters

**DOI:** 10.3390/ijms23158474

**Published:** 2022-07-30

**Authors:** Charles Joussain, Olivier Le Coz, Andrey Pichugin, Peggy Marconi, Filip Lim, Mariaconcetta Sicurella, Keith Foster, François Giuliano, Alberto L. Epstein, Alejandro Aranda Muñoz

**Affiliations:** 1 UMR INSERM U1179—Université de Versailles Saint Quentin en Yvelines/Paris Saclay, UFR des Sciences de la Santé Simone Veil, 2, Avenue de la Source de la Bièvre, 78180 Montigny-le-Bretonneux, France; charles.joussain@uvsq.fr (C.J.); olivier.le-coz@uvsq.fr (O.L.C.); karyolog@gmail.com (A.P.); francois.giuliano@uvsq.fr (F.G.); 4lejandro.4randa@gmail.com (A.A.M.); 2 Neuro-Urology R. Poincaré Hospital AP-HP, 104 bvd R. Poincaré, 92380 Garches, France; 3 Ipsen Innovation SAS, 5 Avenue du Canada, Zone Industrielle de Courtaboeuf, 91940 Les Ulis, France; 4 Department of Chemical, Pharmaceutical and Agricultural Sciences (DOCPAS), Via Luigi Borsari, 44121 Ferrara, Italy; peggy.marconi@unife.it (P.M.); scrmcn@unife.it (M.S.); 5 Centro de Biologia Molecular Severo Ochoa, Universidad Autonoma de Madrid (UAM), CSIC-UAM, Calle Nicolas Cabrera 1, Cantoblanco, 28049 Madrid, Spain; filip.lim@uam.es; 6 IRCCS Ospedale San Raffaele, Urological Research Institute, Via Olgettina 60, 20132 Milan, Italy; 7 Ipsen Bioinnovation Ltd., 102 Park Drive, Milton Park, Abingdon OX14 4RY, UK; drkafoster@gmail.com; 8 EG427, Pépinière Hôpital Cochin, 29 Rue du Faubourg Saint-Jacques, 75014 Paris, France

**Keywords:** HSV-1 amplicon vectors, specific promoter, sensory neurons, peripheral ganglia, organotypic cultures

## Abstract

Background: Neurogenic detrusor overactivity (NDO) is a severe pathological condition characterized by involuntary detrusor contractions leading to urine leakage. This condition is frequent after spinal cord injury (SCI). Gene therapy for NDO requires the development of vectors that express therapeutic transgenes driven by sensory neuron-specific promoters. The aim of this study was to develop and assess tools for the characterization of sensory neuron-specific promoters in dorsal root ganglia (DRG) neurons after transduction with herpes simplex virus type 1 (HSV-1)-based amplicon defective vectors. Methods: The HSV-1 vector genome encoded two independent transcription cassettes: one expressed firefly luciferase (FLuc) driven by different promoters’ candidates (rTRPV1, rASIC3, rCGRP, or hCGRP), and the other expressed a reporter gene driven by an invariable promoter. The strength and selectivity of promoters was assessed in organotypic cultures of explanted adult DRG, or sympathetic and parasympathetic ganglia from control and SCI rats. Results: The rCGRP promoter induced selective expression in the DRG of normal rats. The rTRPV-1 promoter, which did not display selective activity in control rats, induced selective expression in DRG explanted from SCI rats. Conclusions: This study provides a methodology to assess sensory neuron-specific promoters, opening new perspectives for future gene therapy for NDO.

## 1. Introduction

Spinal cord injury (SCI) remains a devastating condition that mainly affects young people; the incidence is around 40 new cases per million people throughout the world each year [1]. Of all the impairments caused by SCI, one of the most important for affected individuals is lower urinary tract dysfunction [2]. About 80 percent of people with SCI have episodes of urinary incontinence, mostly resulting from neurogenic detrusor overactivity (NDO) [3]. NDO can cause urological complications, including renal failure, and significantly impacts quality of life [4]. Current therapies (antimuscarinics and botulinum-toxinA) are initially effective; however, they cause adverse effects and their efficacy reduces overtime [5,6].

The pathophysiology of NDO has been well characterized: it results from the emergence of archaic spinal reflex pathways and the resurgence of neonatal perineal-to-bladder and bladder-to-bladder excitatory reflexes. After SCI, silent nociceptive C-fiber bladder neurons switch to mechanosensitive neurons [7]; this is a key mechanism in the development of NDO.

The Brindley procedure targets the pathophysiology of NDO. It involves neurosurgical sacral deafferentation of the posterior roots that innervate the bladder, combined with stimulation of the anterior roots using an implanted stimulator to trigger micturition on demand and without catheterization [8]. Despite its effectiveness, this procedure is not commonly used [8]. The lack of selectivity of sacral deafferentation is responsible for the loss of remaining pelvi-perineal reflexes, e.g., erection and ejaculation in males and lubrication in females. In addition, the Brindley procedure cannot be proposed in the case of incomplete spinal cord lesion.

Innovative therapies are therefore needed to prevent urological and renal complications and to improve the long-term quality of life in people with NDO. Gene therapy that specifically targets C-fiber bladder afferents is a promising approach for the treatment of NDO. To be clinically effective, such an approach should deliver a transgene that is capable of long-term NDO inhibition by selective expression in bladder sensory neurons. Furthermore, the autonomic efferent neurons (parasympathetic) that represent the motor output of the spinal reflex should be spared so that micturition can be triggered by an implantable electronic device, similarly to the Brindley procedure, thus avoiding catheterization. The non-replicative herpes simplex virus type 1 (HSV-1)-based vector seems be the best candidate for this purpose because: (i) it can establish a persistent non-pathogenic latent infection in sensory neurons, (ii) it has a large genomic capacity (152 kbp) for the construction of vectors that carry large regulatory DNA sequences that support expression from sensory neuron-selective promoters, and (iii) its safety has previously been demonstrated in clinical trials [9].

The aim of this work was to develop tools for the identification of potentially selective promoters that can preferentially express therapeutic transgenes in sensory neurons rather than autonomic neurons. For this purpose, we constructed HSV-1 amplicon vectors that encode a reporter transgene (firefly luciferase, FLuc) driven by different sensory neuron-specific promoter candidates, in addition to an internal control reporter transgene (RLuc-GFP) driven by the nonspecific, ubiquitous HSV-1 IE4/5 promoter. We assessed our promoter candidates in organotypic cultures of dorsal root ganglia (DRG) and autonomic ganglia.

## 2. Results

### 2.1. Construction of Amplicon Vectors

**Figure 1:**
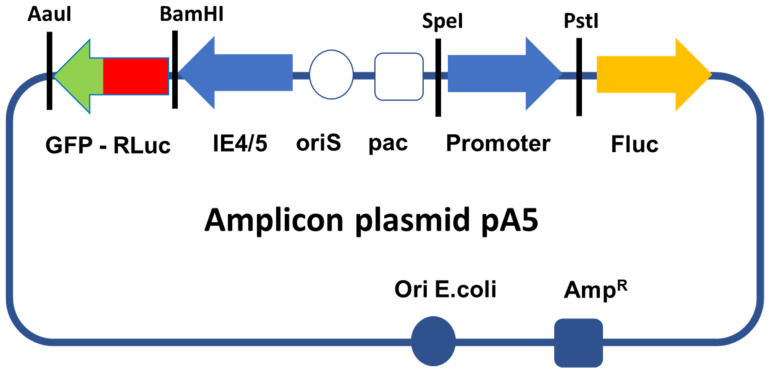
HSV-1-based amplicon plasmids used in this study. The scheme shows the general structure of the amplicon plasmids: oriS and pac (white circle and square respectively) correspond to the origin of viral DNA replication and the packaging sequences of HSV-1, both of which are required to generate vector particles. The blue left-arrow represents the IE4/5 promoter sequence, an HSV-1 immediate-early promoter that drives expression of the RLuc-GFP fusion reporter protein. This transcription unit is terminated by the bovine growth hormone polyadenylation signal. The blue right-arrow corresponds to the different promoter candidates, or to the control E1Fα promoter to be tested, all of which drive the expression of FLuc (orange arrow). This transcription unit is terminated by the SV40 polyadenylation signal. Each amplicon plasmid also contains the gene that confers ampicillin resistance (AmpR, blue square) and an *E. coli* DNA replication origin (blue circle) that allows the plasmid to be amplified in bacteria. Each amplicon plasmid (and the corresponding amplicon vector) therefore carries 2 different independent transcription units: one expresses FLuc driven by a sensory neuron-selective or control promoter candidate, and the other expresses the fused RLuc-GFP protein driven by the viral IE4/5 promoter. The GFP moiety of the RLuc-GFP protein allows vector titration by counting green infected cells. The RLuc moiety of the protein serves as an internal invariable control; it compensates for differences in the number of infected neurons and can be used as a comparator to evaluate the strength of the different selective promoter candidates. Thus, based on the ratio of FLuc/RLuc, these constructs allow the assessment of both the relative strength of different promoters in a same cell type or ganglia, and the tissue selectivity of a given promoter in different ganglia.

We generated a family of amplicon plasmids (the pA5 family) that express firefly luciferase (FLuc) under the control of rTRPV-1, rCGRP, and rASIC3 sensory neuron-specific promoters (rat promoters). The selection of these promoters was based on the results of a non-systematic literature review [7,10,11,12,13,14,15,16,17,18,19] that we conducted to identify proteins expressed in sensory neurons that innervate the bladder, but not (or weakly) expressed in parasympathetic or sympathetic neurons. The relative expression of these proteins should increase in sensory neurons after SCI. In addition, we also selected a sensory neuron promoter of human origin (hCGRP) in a translational perspective. A further vector, which expressed FLuc driven by the EF1α promoter (a strong and non-selective human promoter), was used as a negative control. Figure 1 shows the structure of the amplicon plasmids used to generate the corresponding amplicon vectors.

### 2.2. Establishment of Optimized DRG Organotypic Cultures

**Figure 2:**
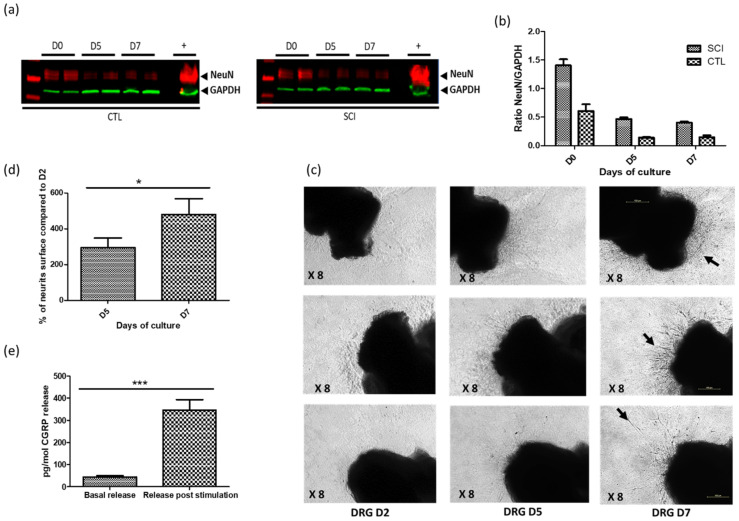
Validation of adult rat organotypic dorsal root ganglia (DRG) cultures. (**a**) Western blot to detect NeuN and GAPDH expression in organotypic DRG cultures at day 0, day 5 and 7 (D0, D5, D7), from control (CTL) and SCI rats (n = 2) + is the positive control (rat’s brain). (**b**) Neuronal survival in DRG cultures prepared from spinalized (SCI) and non-spinalized control (CTL) rats assessed by semiquantitative Western blot, with NeuN expression normalized with respect to GAPDH; initial neuronal death (up to day 5) was followed by stability (from days 5 to 7 (n = 2)). (**c**) Neurite outgrowth in representative DRG cultures from 2 to 7 days of culture (D2, D5, D7). Arrows indicate neurites growth after 7 days of cultures. (**d**) Neurite length and density (manually calculated) increased significantly from day 5 (D5) to day 7 (D7). (**e**) CGRP release by the adult rat organotypic DRG cultures at 3 days of culture, with (release post stimulation) or without (basal release) stimulation with 75 mM of KCl (n = 8) for 30 min. The Student *t* test was performed to assess differences between conditions: * *p* < 0.05, and *** *p* < 0.0001.

To assess the selectivity of vector expression driven by putative sensory neuron-selective promoters, we set up optimized models of organotypic cultures of autonomous ganglia and DRG. The purpose of these models was to allow peripheral infection through the neurites, thus mimicking the natural infection process. Despite initial neuronal death (about 70%) between days 1 and 5 post-culture, the organotypic DRG cultures from both the control and SCI rats then remained stable until at least day 7 (Figure 2a). Moreover, the ganglia displayed time-dependent neurite growth (Figure 2b,c) and a persistent ability to release CGRP neuro-mediator from day 3 to day 6 post-culture (Figure 2d). The survival rates of the organotypic DRG cultures from the SCI rats was comparable, or even better, than those from the control rats at all the post-culture time points (Figure 2a).

### 2.3. Gene Transfer in Organotypic DRG Cultures by HSV-1-Based Vectors

**Figure 3:**
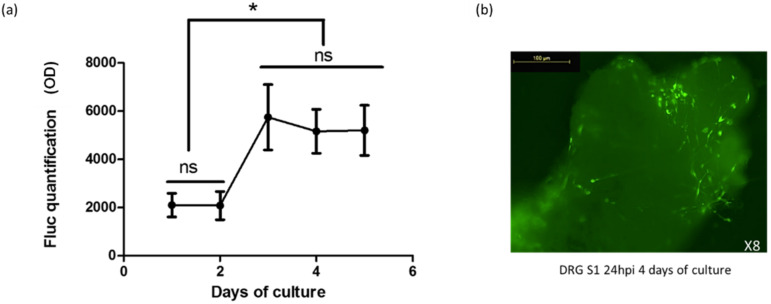
Transduction of adult rat organotypic dorsal root ganglion (DRG) cultures by HSV-1 amplicon vectors. (**a**) Fluc expression 24 h after infection with 10^6^ vector particles (n = 4, blank OD was 450). The background FLuc expression was 450 OD. Fluc expression increased significantly from day 3: Fluc quantifications for data at day 1 and 2 were significantly different from each value at day 3, 4 and 5. The Student *t* test was performed to assess differences in transgene expression between these ganglia; ns: not significant; *: *p* < 0.05. (**b**) Transduction of DRG S1 neurons with HSV-1 amplicons expressing GFP. Ganglia at day 4 of culture were analyzed by fluorescence microscopy 24 h after infection (24 hpi). OD: optical density.

Our aim was to develop a model of organotypic culture of DRG that reproduced the natural infection pathway of HSV-1: penetration at the terminal ends followed by retrograde transport of the virus particles to the neuron nucleus, located in the soma of the ganglion. Since the growth of neurites is time dependent, organotypic DRG cultures were infected with vectors at different post-culture time points, to determine infection timing (Figure 3). Infection levels were assessed by luciferase assay performed one day post infection.

The organotypic DRG cultures increased by three times its infection rate on day 3 post-culture. At this time, neurites had emerged from the ganglia and a significant increase in FLuc expression could be detected (Figure 3). Infection rates then remained stable between days 3 and 5 post-culture. Figure 3b shows intra-ganglia infected cells, 24 h post-infection at day 4 post-culture. Therefore, considering both neuronal survival and infectibility, we suggest that day 3 to 4 post-culture is the best time to infect ganglion sensory neurons.

### 2.4. Selective Expression of RCGRP Promoter in Adult Rat DRG Ganglia

**Figure 4:**
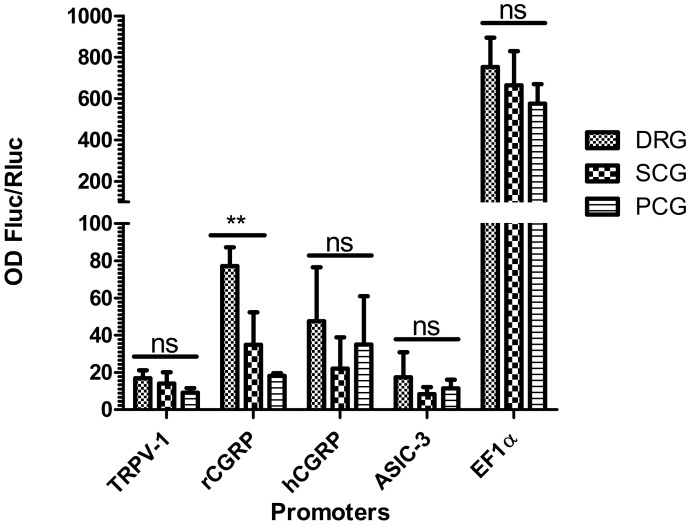
Organotypic neuronal cultures of adult rat dorsal root ganglia (DRG), superior cervical ganglia (SCG), and paracervical ganglia (PCG) were infected after 3 days in culture with 2.10^6^ vector particles, and protein extracts were prepared after 24 h for analysis of luciferase activity. The histogram bars show normalized ratios (FLuc/RLuc, n = 3 per promoter and per ganglion type), which quantify the activity of Fluc driven by a candidate promoter relative to the activity of RLuc (in the RLuc-GFP fusion protein), which is driven by the constant ubiquitous HSV-1 IE4/5 promoter. The ANOVA test was performed to assess differences in transgene expression between these ganglia; ns: not significant; ** *p* < 0.001. Of the candidate promoters, only rCGRP appeared to be selective for DRG sensory neurons. The control EF1α promoter showed no selectivity, as expected. OD: optical density.

To assess the selectivity of different sensory neuron-selective promoter candidates, we assessed vector constructs that expressed FLuc and RLuc-GFP in organotypic cultures of autonomic ganglia and DRG from adult control rats. A vector encoding FLuc driven by the cellular EF1α promoter was used as a nonspecific control (Figure 4). Expression driven by sensory neuron-selective promoter candidates was much lower than that driven by the EF1α promoter. Of the promoter candidates, only rCGRP displayed significant selectivity for DRG neurons (assessed by FLuc/RLuc expression). All the other promoters displayed a trend for DRG selectivity but differences among the various ganglion cultures were not statistically significant (Figure 4). Interestingly, although the rCGRP promoter displayed selectivity for DRG, the hCGRP promoter did not.

### 2.5. Selective Expression of the RTRPV-1 Promoter in DRG Ganglia from SCI Rats

**Figure 5:**
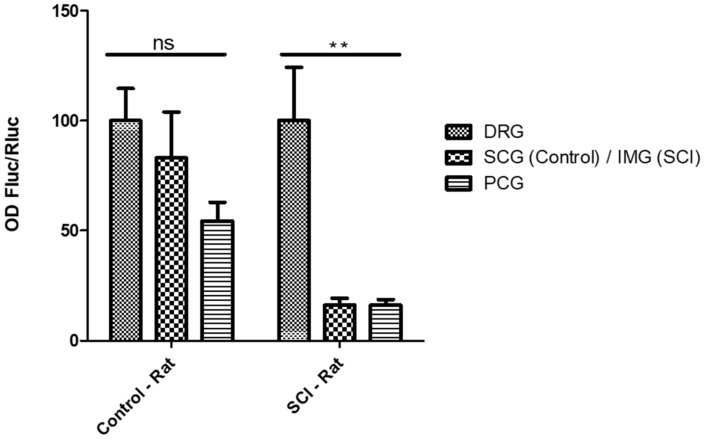
Organotypic neuronal cultures of adult rat dorsal root ganglia (DRG), superior cervical ganglia (SCG), and paracervical ganglia (PCG) were infected after 3 days in culture with 1.10^6^ particles of vector expressing FLuc driven by the TRPV-1 promoter, and protein extracts were prepared after 24 h for analysis of luciferase activity. The histogram bars show normalized ratios (FLuc/RLuc, n = 3 per promoter and per ganglion type), which quantify the activity of Fluc driven by a candidate promoter relative to the activity of RLuc (in the RLuc-GFP fusion protein), which is driven by the constant ubiquitous HSV-1 IE4/5 promoter. Results are presented as percentages of DRG values, considered to be 100%. Data from control rats are those previously shown in Figure 4. The ANOVA test was performed to assess differences in transgene expression between these ganglia, ns: not significant; ** *p* < 0.001. In organotypic cultures from SCI rats, the TRPV-1 promoter exhibited significant selective activity compared to that in autonomous ganglia that innervate the bladder. OD: optical density. IMG: inferior mesenteric ganglion.

The TRPV-1 promoter did not demonstrate significant selective expression in the DRG from control rats. Therefore, to assess if intralesional plasticity following SCI can modify the activity of this promoter by increasing its selectivity for DRG, we infected bladder peripheral ganglia from both control and SCI rats with vectors that encoded this promoter. We used infra-mesenteric sympathetic ganglia for this study because they are located below the level of the spinal lesion and would thus be impacted by any neuronal plasticity that occurred following SCI. The TRPV-1 promoter exhibited selective expression in the organotypic cultures of DRG explanted from rats with SCI, but not from the control rats (Figure 5).

## 3. Discussion

This study demonstrates the viability of DRG organotypic cultures with a stable infection rate with HSV-1 amplicon vectors coding for two expression cassettes. This allowed us to quantify the strength and selectivity of sensory neuron-specific promoter candidates.

Our aim was to create a gene therapy to treat NDO in individuals with paraplegia and tetraplegia that allows micturition to be triggered on demand. Our strategy is based on the silencing of bladder DRG neurons through the selective expression of therapeutic transgenes mainly in these neurons, thus sparing the motor output of the micturition spinal reflex and allowing on-demand electric stimulation. HSV-1 mainly establishes latent infections in sensory neurons; however, the regulatory sequences that drive the expression of the latency associated transcripts (LATs) can also be expressed in latent infections in autonomic neurons [20,21]. By comparison, several studies have demonstrated that tissue-selective expression from HSV-1 vectors can be obtained both in neurons and in non-neuronal cells in vivo and in vitro by placing the transgenes under the control of tissue-specific promoters [22,23,24].

It was critical, therefore, to identify sensory neuron-specific promoters displaying selective expression in sensory neurons, and for this we developed tools to assess the expression of several promoter candidates when delivered to the different peripheral ganglia via the genome of replication-defective HSV-1 amplicon vectors. We generated a set of amplicon vectors in which the expression of a reporter protein (FLuc) is driven by some of the selected promoters (rTRPV-1, rASIC3, and rCGRP). We also constructed and studied a vector in which FLuc is driven by the human hCGRP promoter, from a translational perspective. The activity of these vectors was evaluated using organotypic cultures of sensory and autonomic ganglia, thus decreasing the number of animals used, in line with the new international guidelines for animal experimental procedures [25].

Despite an initially high level of neuronal death, neuron viability remained stable for enough time for neurite outgrowth to occur. Moreover, at 7 days post-culture, the ganglia still released CGRP, indicating that peptidergic fibers, including C-fibers, which are involved in NDO, remained functional. The ganglia only became infected by the vectors after neurite outgrowth, corresponding to the natural infection process in which HSV-1 enters neurons through their extremities. This finding validates the use of optimized ganglia organotypic cultures to assess the selectivity of expression of sensory neuron-selective promoter candidates. Our results indicate that the promoters tested are much weaker than the control EF1α promoter in peripheral neurons. Only the rCGRP promoter displayed selective expression in the DRG. In contrast to rCGRP, hCGRP did not exhibit increased expression in rat DRG compared to rat autonomic ganglia, a finding probably related to species selectivity. Indeed, the very low homology between rat and human promoters and potential differences in the associated transcription factors can explain the low selectivity of hCGRP promoter in rat tissue. Finally, we compared expression of the TRPV-1 promoter both in control and SCI rats. The TRPV-1 promoter did not display any selectivity in ganglia from control rats; however, it was selectively expressed in the DRG from SCI rats, thus indicating overexpression in the DRG and a possible reduction in expression in autonomic ganglia following spinalization. This finding is supported by a recent study that demonstrated that TRPV-1 and CGRP promoters allowed effective transgene expression in transduced sensory mice neurons following in vivo intravesical delivery, with an increase in transgene expression following spinalization [10]. The increased selectivity of expression of the TRPV-1 promoter in SCI rats may have resulted from an increase in the density and size of C-fibers following spinalization [10]. However, as no other ganglia were included in that study, the selectivity of these promoters for DRG could not be evaluated. Finally, the transduction of satellite glial cells in the DRG can lead to a greater expression of luciferase under the control of TRPV-1 or ASIC promoters, as the corresponding proteins are known to be expressed in satellite glial cells. However, the expression of luciferase driven by these promoters did not differ significantly in terms of selectivity in the non-SCI rats. In contrast, satellite glial cells do not express CGRP, whereas luciferase expression driven by rCGRP was significantly higher in the DRG.

To our knowledge, our study is the first to evaluate the selectivity of sensory neuron-selective promoters in sensory versus autonomic ganglia, allowing the identification of a candidate for the development of potential therapeutic vectors.

We recently demonstrated the efficacy of defective HSV-1 vectors expressing botulinum neurotoxin light chains (BoNT-LC) to cleave SNARE proteins, thereby inhibiting CGRP release and disrupting neurotransmission [26]. Based on these results, in addition to those reported here, we are currently generating novel vectors that express BoNT-LC driven by rCGRP or hCGRP to determine whether they display sensory neuron-selective transgene expression in primary cultures of both rat and human sensory neurons. The use of a human sensory neuron-selective promoter assessed in human tissues will reinforce the validity of these constructions, thereby providing new perspectives for the development of therapies to treat NDO.

## 4. Materials and Methods

### 4.1. Organotypic Cultures of Adult Rat Ganglia

Organotypic cultures were prepared from ganglia from 2 types of rat: healthy, adult rats (250 g) and spinalized adult rats. All were female Sprague–Dawley rats. The spinalized rats were sacrificed 14 days after they had undergone a T8–T9 spinal cord transection [27]. Euthanasia was performed by progressively increasing concentrations of CO_2_ in accordance with the European Communities Council Directives 86/609/EEC on the use of laboratory animal and care regulation in force in France (Ministry of Agriculture, Authorization Agreement No. A78-322-3, December 2013 and B78-423-1 July 2017). After euthanasia, ganglia were harvested.

Rat DRG were collected as follows: a dorsal middle incision was performed to expose the S3-L4 vertebrae. A laminectomy was then performed to expose the spinal cord and DRG from S2 to L4. The S1 and L6 DRG, which are bladder-specific, were collected separately.

Paracervical ganglia (PCG), which are parasympathetic, were collected as follows: laparotomy was performed and the perimetrium connective tissue was exposed and dissected laterally and slightly posteriorly to the vagina-uterine cervix junction where PCG were collected.

Infra-mesenteric ganglion (IMG) and superior cervical ganglia (SCG), both of which are sympathetic, were collected as follows: laparotomy was performed to expose the abdominal aorta. The inferior mesenteric artery was identified and the IMG, which lie on this artery, was collected. To collect SCG, the animals were beheaded and the tissues were dissected to expose the common carotid artery until the carotid bifurcation where the SCG are located, after which the ganglia were collected.

Organotypic cultures were set up according to a protocol adapted from Lin et al. [28]. In all cases, collected ganglia were immediately placed in a 24-well plate precoated with 15 to 20 µL of Matrigel (SIGMA, Saint Louis, MO, USA) 30 min before plating. The ganglia were cultured as follows: after a 30 min incubation period, 500 µL of Neurobasal Sup Medium (Neurobasal medium [Gibco^®^ by life Technologies, 21103-049, Grand Island, NY, USA], 5–10% inactivated fetal bovine serum, 2% B27 [Gibco^®^, 17504-044, Grand Island, NY, USA], 2 mM L-glutamine [Gibco^®^ by life Technologies, 25030-032, Grand Island, NY, USA], 50 U/mL streptomycin and penicillin and 250 ng/mL NGF [Sigma N2513-1MG, Saint Louis, MO, USA]) was added to the culture. The medium was changed every 2 days.

### 4.2. Construction and Titration of Non-Replicative HSV-1 Amplicon Vectors

(a) Plasmids containing sensory neuron-specific promoters. A family of pUC57-proms plasmids carrying the sequences of 4 promoters that were expected to be selectively expressed in sensory neurons, rat TRPV-1, rat ASIC3, rat CGRP, human CGRP, and EF1α as a control non-selective promoter, was ordered from GenScript Biotech Corps, Piscataway, NJ, USA (Gene ID numbers: rTRPV-1: 83810, rASIC3: 286920, rCGRP: 24241, hCGRP: 796, EF1α: 1915, see Supplementary Materials). All the sequences were engineered to contain a SpeI site at the 5′ end, and PstI (hCGRP, EF1α) or NsiI (TRPV-1, rCGRP, ASIC-3) sites at the 3′ end to facilitate sub-cloning into amplicon plasmids.
(b) Generation of amplicon plasmids. A pUC57 plasmid carrying the sequence of renilla luciferase (RLuc) and fused to a green fluorescent protein (GFP) through an alpha helix (RLuc-GFP, 5′Rluc-αH-GFP3′) was also ordered from GenScript. This sequence, which is flanked by a BamHI site in 5′ and an AauI site in 3′, was sub-cloned into a pGemT-easy plasmid, thus producing plasmid pG-RLuc-GFP. To generate the amplicon vectors used in this study, we modified a previously described amplicon plasmid pA-EUA2-Luc [28]. This plasmid carries 2 independent transcription cassettes: one that expresses GFP driven by an HSV-1 immediate-early promoter (IE4/5), and one that expresses firefly luciferase (Fluc) driven by the immediate early human cytomegalovirus (HCMV) promoter. We first digested pA-EAU2-Luc and pG-RLuc-GFP with BamHI and AauI to delete the GFP open reading frame from pA-EUA2-Luc and replaced this sequence by the RLuc-GFP sequence from pG-RLuc-GFP, thus creating the amplicon plasmid pA-RLuc-GFP. Then, plasmid pA-RLuc-GFP was digested by SpeI and PstI to delete the HCMV promoter, which was replaced by each of the promoters from the pUC57-proms plasmids, thus creating the 5 members of the pA5 family of amplicon plasmids, pA-rTRPV-1-Luc, pA-rASIC3-1-Luc, pA-rCGRP-Luc, pA-hCGRP-Luc, and pA-EF1α -Luc, which express RLuc-GFP driven by the IE4/5 promoter and Fluc driven by each of the different sensory neuron selective promoters (Figure 1).
(c) Production and titration of amplicon vector particles. Amplicon vector stocks were prepared and titrated as previously described [28,29,30]. Cells expressing fluorescent GFP were counted at 24 h post-infection using an inverted fluorescence microscope (Olympus, Tokyo, Japan) with transducing units (TU). Titers of helper virus particles were provided as plaque-forming units (PFU) per milliliter [29,30]. Serial passages of the vector populations were then carried out on 7b cells, thus increasing the TU/PFU ratio to 99/1 or higher.

### 4.3. Infection and Harvesting

At 3 days post-culture, neurite outgrowth had occurred; thus, the neurons from the organotypic ganglia cultures were more susceptible to infection from the vectors. At this time, 5 × 10^6^ infectious particles were pipetted directly onto the ganglia and the cultures were kept overnight at 37 °C with 5% CO_2_. The ganglia were harvested 24 h post-infection. The medium was removed and 100 µL of Dispase (Sigma, Saint Louis, MO, USA) was added over 1 h to dissolve the Matrigel and release the ganglia. The ganglia were then collected and conserved at −80° until the luciferase assays were performed.

### 4.4. Western Blots (WB)

The ganglia were mechanically dissociated using Precellys 24 Bertin technologies into a RIPA lysis buffer (Thermofisher, Rockford, IL, USA). After tissue lysis, homogenates were centrifuged (4 °C, 10,000 rpm) for 10 min. Pellets were resuspended in a Laemmli buffer (Bio-Rad). After protein quantification, 50 µg of protein was used to perform electrophoresis (Tris-HCl PAGE-Gel (Bio-Rad laboratories Inc., Hercules, CA, USA). The transfer of proteins was performed using an activated-methanol membrane (0.35 A) (Merck Millipore, Tullagreen, Carrigtwohill, Co, Cork, Ireland, Immobilon FL Transfer Membrane) at 4 °C for 90 min. Blocking and antibody incubation were performed using the IBindTM Flex Western System SLF2000 (Invitrogen). The intensity of each band was determined by pixel density integration using LI-COR**^®^** Odyssey Western Blotting Kits. Antibodies used are presented in Table 1.

### 4.5. Luciferase Quantification

Luciferase quantification was performed using a Dual-Luciferase**^®^** Reporter Assay System (Promega). Ganglia were disrupted by placing them into a Lysing matrix D-Tube-RNAse/DNAse free Eppendorf (MP Biomedicals) with 200 µL of passive lysis buffer (Precellys 24 Bertin technologies). The tissue lysate was centrifuged for 15 min at 4 °C and 12,000 rpm. Then, 20 µL of supernatant was deposed in a 96-well black plate (Nunc #: 237107) in which 100 µL of LARII buffer (Promega) was added. In a second step, 100 µL of Stop and Glo buffer (Promega) was added to inhibit FLuc expression, and to allow RLuc expression. Luciferase expressions were immediately quantified using a luminometer (FLUOstar Omega BMG LABTECH, France). Promoter strength in any specific tissue was determined by the ratio between the FLuc and RLuc, corresponding to transgene expression normalized by the rate of infection.

### 4.6. Statistics

Data were described by the mean and SEM values. Student *t* test and ANOVA test were used, and *p* < 0.05 was considered statistically significant. GraphPad Prism v5, San Diego, CA, USA was used for all analyses.

### 4.7. Ethics

All genetic constructs used in this study were declared as genetically modified organisms to the French regulatory agency (authorization file N° 3910) and the study was conducted in accordance with the safety and confinement requirements established by the French regulatory authorities.

## 5. Patents

This study is associated with the following patent: WO2017220800A1.

## Figures and Tables

**Table 1:** Antibodies used in this study.

Primary Antibodies	Brand	Reference	Species	Dilution
GAPDH	Millipore	CB1001	Mouse	3/4000
NeuN	Abcam	ab177487	Rabbit	1/400
Secondary Antibodies	Brand	Reference	Species	
Mouse (800)	Li-Cor	926-32210	Goat	3/4000
Rabbit (680)	Li-Cor	926-68071	Goat	3/4000

## Data Availability

Not applicable.

## Supplementary Materials

The following supporting information can be downloaded at: https://www.mdpi.com/article/10.3390/ijms23158474/s1.
