# Supplementary material for: Development and Assessment of Herpes Simplex Virus Type 1 (HSV-1) Amplicon Vectors with Sensory Neuron-Selective Promoters
Source: Int J Mol Sci. 2022 Jul 30;23(15):8474. doi: 10.3390/ijms23158474 (PMC9369297; doi:10.3390/ijms23158474)
Supplement: Supplementary file 1 [file ijms-23-08474-s001.zip › ijms-1805943-supplementary.pdf]

International Journal of
Molecular Sciences

MDPI

Supplementary Materials

TRPV1

actagtatatggagaggtggggtgaggggtggcagagagggatcgagagaggagagagggaaccagatgtagcagccaggaggccaaaggtacaaaaggggtgggtaaccaaaatgt
ctggattatataaaaaagagccagaggtcaggcccactttgatatgttaaataggcacctcagccatttatccaggtttgaaatgtaatataatttacatccccctggcttcctagagaccgttgtttag
acggatgacctctgcagaatgtttgagggtgcagtcttgcatgtactccctggtgggctttcttgggcaggatctgggcaggaatgggcttgttctagtcacccactgcgtatgatggatgaaccc
gcttcctagtagttaggatggcactgggggaggcgagaaattagcacacgtaacgttttcttgtgttctattgttcactaagggaccccagtcaagcaagactgggccttggaagacctagagacc
accaaacctaatctctaccccgggtctgagtacacagggactcagagtcccaaagggggcagggcctccagacaggtggctcagaggtcccagtcctttggaaacatggcatcttcaggacact
gggctttgcatctctggctgtgacagtcctttaagggagctactcctcagacatacaggagagatggtttggaaagtccgagatccaaagcctggttcaggctggactgggctgcaggctgctaa
gtgctcctctgccctggcatggctgggggtggggcattggctgtggtttctgaaaaagggcaaaaatgatgggaaaagctttgggatcctctgggaatcggagccgtggtaacagcagctgct
gccattgctgcaaatgtttccttgagtgccagagtatgcccagagcccatccctgccgtacgccaggggaggggcgaggaccctcacagaggcagggaggccggccactcttaccacacagca
gcctggctctcccacaaagaacagctatgcat

rCGRP

actagtctgcaggagagggacttgaaggcaggcttgggacaatttgagaatgaacccctaaggatgcttctgtgggccacagagactgctgagtggctgtgctttctggatacggtaccattttgga
aagagaggagagtctctgtgccaggagaagtgtgactggtagtgaatgtgaggtttagtacggggcaacatctccacagcgctgtcaagcctgcctgcctgctcttcagctctttagctcggaga
tctaagggtgggggtaggaggggagccaccggaccaaatacaactgggacatcttggcaaacagcagcgggaagcaaaggggcagctgtgcaaatccttaggcaggcgggcgggcggg
caggcgggcgggcgggcgggcaggcgggcaggcgggcgggcaggcggccggatgagtagtgatggatagccaggcaggaggtggagagatctacactggagactttagaggcatctg
gtccttcctcacactgtccccactaccccgtaccctactccctaccccaagcaggacccagctgaatacaaccccttctcacacatgtgagtgagtgagttatccagcacataagaatgccaagctg
aagacggatgattcactttggggaaggagagatttttatagctcaggaaacaccaaggtttctgcctactagccaggcccttcaaaaggggaccaggatacccactgaaaagtttaatatgttgag
ctttcgtgcaggcctttggggggtttggggggggggggaattttgaatttttttttcgttttgtttttacctgtggtcacataaccagcacgaggcagctacaaggttcaggtctgacagagcccctgtgt
ccagcaccaacacctttggctatcagcctaaacctgtgccaccctgccaaagccagccttgcagaccaagagtccacccctacggtgcactaaagtcttccggattaggcacggactagggtcgg
ggcacgattagaatcagacatgcagcaaggagtacttgagatactggactctactctccaaggtccagagattggagtcggggatgttcaaagtcaggagggaagaagagataaaatttacctt
gacgtcaaaaggccctccaaattcccgctaattttaagggtggttctcactgctccccaccatcctcccacttccatcaatgacctcaatttaaattcaaatggtgtcatcttgctagatgctcggagttc
tggaagcaccgaggtgacgcaatctgtctggggcacggggcccttccacctattggctgcctggcgccccgggacccctcccaactaccgcggcgggaataagagcagctgcaggcgcttgg
aaatgcat

hCGRP

actagtgatcaattaagggcatcttagaagttaggcgttcccgctgcctcctttgagcacggaggccaccaaccccctagggggaagagatgtagcgcgaggcaggggtgtcgtgctaagaaa
tttcgacgcttctggggactgaggacaaaggtgcggacacgaccccggggtacctggagttccgtgactcgcgccacggacggcacacctaggggctaatttctgctctgcctcaaagaacctc
aagctagagtccttgcctccgcccacagccccgggatgccgctgctgcgctcaccgcacaggcagcgcccggaccggctgcagcagatcgcgcgctgcgcgttccaccgggagatggtggag
acgctgaaaagcttctttcttgccactctggacgctgtgggcggcaagcgccttagtccctacctctgctgagctgaacgctcaggcacagtggaactgaaacccggttctgcgggatgtgagag
ctgttgaggtcacgcgtaattgggtgtgatggagggcgcctgttcgtgatgtgtgcaggtttgatgcaagcaggtcatcgtcgtgcgagtgtgtggatgcgaccgcccgagagactcggaggc
aggcttgggacacgtttgagtgaacacctcaggatactcttctggccagtatctgttttttagtgtctgtgattcagagtgggcacatgttgggagacagtaatgggtttgggtgtgtgtaaatgagt
gtgaccggaagcgagtgtgagcttgatctaggcagggaccacacagcactgtcacacctgcctgctctttagtagaggactgaagtgcgggggtgggggtacggggccggaatagaatgtct
ctgggacatcttggcaaacagcagccggaagcaaaggggcagctgtgcaaacggctcaggcaggtgatggatggcagggtaggaagggggaggtccagaggtctggatggaggcttccg
catctgtaccttgcaactcacccctcaggcccagcaggtcatcggccccctcctcacacatgtaatggatctgaagagtaccccgggacagtccggggagatggagattcggaaagtatccatgg
agatcttacagaatcccctatgcggaccaggaaactcttgtagatccctgcctatctgaggcccaggcgctgggctgtttctcacaatattccttcaagatgagattgtggtccccatttcaaagatga
gtacactgagcctctgtgaagttacttgcccatgatcacacaaccaggaattgggccaactgtaattgaactcctgtctaacaaagttcttgctcccagctccgtctcttgtttcccacgagccctggcc
ctctgtgggtaataccagctactggagtcagatttcttgggcccagaacccacccttaggggcattaacctttaaaatctcacttgggcaggggtctgggatcagagttggaagagtccctacaatc
ctggaccctttccgccaaatcgtgaaaccaggggtggagtggggcgagggttcaaaaccaggccggactgagaggtgaaattcaccatgacgtcaaactgccctcaaattcccgctcactttaa
gggcgttacttgttggtgcccccaccatcccccaccatttccatcaatgacctcaatgcaaatacaagtgggacggtcctgctggatcctccaggttctggaagcatgagggtgacgcaacccagg
ggcaaaggacccctccgcccattggttgctgtgcactggcggaactttcccgacccacagcggcgggaataagagcagtcgctggcgctgggaggcctgcag

ASIC3

actagtcctagcctttgtgagagtctgccccaaaggtggaagcactgttaatacaagttgatctgagtaaccataaggatcgtggaagtggtgggatggtgcctcaccttgaacaggggaagca
ggcagcctgtagtcccaaagctggggtctccgttggcttctcaggtgttgagaaaagtacttgaaagatcctgtagactacactgatgctcccagaggtcagccctttgtctactctgctgcagattc
cttgatccacgtcacttaaacccagaggatggtggccccctagccttggtacagtaggtcactctcccactgagaatccatccctggccagccacatccaggttataggactgcagatcagggactg
gatcgggggggaggggctataactgcatgaccagacaagcatttgtacttttcatgtggcaaaggatgacattgtctttcaggagtgattggtgcagaggaccaacagctgctgttgggggccag
tgtatcaccacatacactgctgtgaggacgaggaaaccaagaaccctagtccgtggtgccccggttcagtgtccgtagtgacagcctgggaagcagagctctggggtgacttccactgcctggct
actgcgcccatcccctcccctaacaagggtagcttagagcaaggctttcatggcatgtccctctgtctagccctaccttttctccttcaccgagcatccagaccattggctccacgtacacacagattct
gtccctgacagccacaggatgactgcccatcagggaaggaccccctatggtcagtggccggagaccccaaccagtagctatgtcaccctgtctgtcacctggcctggctttcccagcccctcagag
aacagcgacttgatgactcttttaattctttttttttaaagatttatttatttattttgtatatgagtacactgtagctgtcttctgacacaccagaaagggcatcggattccattacagatggttgtgagcca
ccatgtggttgctgggatttgaactcaggacctctggaagagcagtcggtgctcttaaccgctgagccatctctccagcccagacttgatgactcttgctcttaaaagctcccaagagttaaatgaca
catgttggaattacgcccttaaaaaccacagtaaaatagccccttctgaggctttctagtggtcggtcacatcagtgccacaaacccctttcagttacctatccttcctaagatgttacttgcctagtctgt
gccactaagcctgggaccattcctgagactaagccccagtgcttatgaggagggccgatgtccatccagtcagagccagcacagtggactgattggttggtgggattatgtccccaccactcatg
gccagaagccaccaggacgaacctggcggtctccagtgcttttgacatggggccagtgatattattgctcaacaagtggagaacctgccaccaaacctaaggagccacattcagtcccccaaga
cttgtggatgagcaacctccaaaagttatcctcggacctccacgtgttgtgacacacatacccagtaaatgaataaaaaataaaactagaaaaatttatgcgaatttgtctgattactcttgagcgtac
aagattttgcccaattcctagatacatgaaggtatatgagactgtagcccataccgaacatgacagtgacagaaccaggccttgcagtcccctgggcagatgtccttgagtgactggcccctctcgt

ctgagtcccttgtactccttcctccaagccagctagcaccaagcttatgggtgactcctcctgcactggaagcacacaggtcccatcccccaagctctctcctcatcttcctttcatctgcattcctagaa
gtagcttcaggacctgatcacatgctgcctttgcctcaaggagggcagagtgcaggcgtggagacccctgcaggcacattccctggtcaagactactaggaatgcaaaatggcattcctgctatc
agggtgggagtgggggatctgatgacatcgatcagatgagtaaggcagtgccctcagcccagaaactctgtttcaggcagtttccctatgacatcaatagcaaagctggaaagtccagtggttat
cggagaagacataagacagcacctccagtaatgagggtgcttttcgggttctgaagttcatggtccctagaatatgtttgttgagagaggaaaatgtaaagcagcggagtaccctgaatacctct
gggcattgtgaaggaggacagcttcgctaacaggagggcctgagaatgaggtggggtctgggtctccaggcagagacggatactcagaagacagaaagccacgggatacggaaactaaa
gagagccccggtcacgtagtattgctaggatgctaggggcataagaagatctgcctgtgccaaacctcccaccactctctaacttggtcaaaagccaagtccactctgcaaaagaacaagcgatc
tcaggtagggtcacagaagggcaggagaggcaaaaggagagaggggatagactaagaatcagccgctgaccccaggtctagaattcaggagctctgaccaggagcctaaaaggccccaa
aggcatcccaaacccaaatgtatatctgggtttccttcaagccctaccatggactgctgtggtttcctcctggcgcctctagtctctgacaacagtgactccccccatgctgcattaatggctcagcctg
gggccccagggaccttccctacccccccagactgctctgctgccccccagcccccctcatgcat

EF1α

actagtgtgaggctccggtgcccgtcagtgggcagagcgcacatcgcccacagtccccgagaagttggggggaggggtcggcaattgaaccggtgcctagagaaggtggcgcggggtaaa
ctgggaaagtgatgtcgtgtactggctccgcctttttcccgagggtgggggagaaccgtatataagtgcagtagtcgccgtgaacgttctttttcgcaacgggtttgccgccagaacacaggtaag
tgccgtgtgtggttcccgcgggcctggcctctttacgggttatggcccttgcgtgccttgaattacttccacctggctccagtacgtgattcttgatcccgagctggagccaggggcgggccttgcgc
tttaggagccccttcgcctcgtgcttgagttgaggcctggcctgggcgctggggccgccgcgtgcgaatctggtggcaccttcgcgcctgtctcgctgctttcgataagtctctagccatttaaaattt
ttgatgacctgctgcgacgctttttttctggcaagatagtcttgtaaatgcgggccaggatctgcacactggtatttcggtttttggggccgcgggcggcgacggggcccgtgcgtcccagcgcac
atgttcggcgaggcggggcctgcgagcgcggccaccgagaatcggacgggggtagtctcaagctggccggcctgctctggtgcctggcctcgcgccgccgtgtatcgccccgccctgggcgg
caaggctggcccggtcggcaccagttgcgtgagcggaaagatggccgcttcccggccctgctccagggggctcaaaatggaggacgcggcgctcgggagagcgggcgggtgagtcaccca
cacaaaggaaaggggcctttccgtcctcagccgtcgcttcatgtgactccacggagtaccgggcgccgtccaggcacctcgattagttctggagcttttggagtacgtcgtctttaggttggggggg
aggggttttatgcgatggagtttccccacactgagtgggtggagactgaagttaggccagcttggcacttgatgtaattctccttggaatttgccctttttgagtttggatcttggttcattctcaagcct
cagacagtggttcaaagtttttttcttccatttcaggtgtcgtgaactgcag
